# Copper Replacement: A Life-Saving Treatment

**DOI:** 10.7759/cureus.103621

**Published:** 2026-02-14

**Authors:** Ami J Patel

**Affiliations:** 1 Interventional Cardiology, Sky Lakes Medical Center, Klamath Falls, USA

**Keywords:** anemia, copper deficiency, end-stage renal disease (esrd), gastric bypass surgery, jehovah's witness patient

## Abstract

Anemia is the hematological hallmark of copper deficiency; however, the infrequency with which it is encountered makes copper deficiency a formidable diagnostic challenge. We present the case of a 71-year-old woman who is a Jehovah’s Witness with a history of cardiomyopathy, end-stage renal disease on peritoneal dialysis, prior gastric bypass surgery, and chronic anemia, who presented with generalized weakness. She was initially diagnosed with anemia in 2003, with a hemoglobin level of 10 g/dL. Her anemia progressively worsened over time, prompting a thorough gastroenterology evaluation with no abnormalities and eventually requiring aggressive treatment with oral and parenteral iron. However, she remained persistently anemic, causing a rapid deterioration in her functional status with hemoglobin worsening to 6 g/dL. An extensive diagnostic workup, inclusive of iron studies, B12 levels, and hemolytic panel was unremarkable. Despite receiving 20,000 units of epoetin alfa three times weekly and intravenous iron supplementation, her hemoglobin level failed to improve. Eventually, hematology was consulted, and a copper level was checked, which was significantly low at 38 μg/dL. Her functional status dramatically improved following intravenous copper replenishment, and her hemoglobin increased from 6 g/dL on admission to 10 g/dL in four weeks. This case illustrates the importance of thinking about copper deficiency in a patient with a history of gastric bypass. It could be lifesaving for a patient who is a Jehovah’s Witness.

## Introduction

Copper is an essential trace element required for erythropoiesis, leukocyte maturation, neurological function, and iron metabolism [1,2]. While dietary copper deficiency is rare, acquired deficiency may occur in malabsorption syndromes, prolonged parenteral nutrition, bariatric surgery, or excess zinc therapy [1,2]. Roux-en-Y gastric bypass (RYGB) bypasses the stomach and proximal duodenum, the primary sites of copper absorption, thereby predisposing patients to late-onset micronutrient deficiencies [2-4].

Hematologic manifestations include anemia, neutropenia, and occasionally thrombocytopenia. Copper-deficiency anemia may be microcytic, normocytic, or macrocytic [5,6]. Bone marrow findings often mimic myelodysplastic syndrome (MDS), with vacuolated precursors, ringed sideroblasts, and multilineage dysplasia, potentially leading to misdiagnosis [5]. Neurologic complications such as gait instability, myelopathy, and peripheral neuropathy may also occur.

Timely recognition is critical, especially in patients who cannot receive transfusions, such as Jehovah’s Witnesses. We present a case of severe, transfusion-refractory anemia due to copper deficiency occurring years after gastric bypass, emphasizing the dramatic hematologic response to copper replacement.

## Case presentation

A 71-year-old female with nonischemic cardiomyopathy (ejection fraction {EF}: 35%), atrial fibrillation with sick sinus syndrome status post dual-chamber pacemaker, mitral valve repair in 2014, type 2 diabetes mellitus, end-stage renal disease on peritoneal dialysis, and prior Roux-en-Y gastric bypass (RYGB) presented with progressive generalized weakness. Her baseline hemoglobin had been ~10 g/dL since 2003.

She was initially diagnosed with normocytic anemia in 2003 and underwent bone marrow biopsy, which was unremarkable. Extensive gastrointestinal evaluation, including esophagogastroduodenoscopy, colonoscopy, and video capsule endoscopy, showed no abnormalities. Her anemia was attributed to chronic kidney disease and treated with oral and IV iron and erythropoiesis-stimulating agents [3,4]. As a Jehovah’s Witness, she refused blood transfusions.

On presentation, she reported profound fatigue and inability to ambulate independently. Examination revealed conjunctival pallor. Admission vital signs and laboratory investigations are summarized in Table 1. Laboratory studies demonstrated a hemoglobin level of 6.0 g/dL with normal MCV. Iron studies revealed adequate iron stores with elevated ferritin, B12 was 1,713 pg/mL, folate was >2,684 pg/mL, and normal hemolysis markers, excluding megaloblastic anemia or hemolysis [5].

**Table 1:** Vital signs and laboratory evaluation on admission. This table summarizes the vital signs and laboratory results at the time of admission, including complete blood count, iron studies, nutritional markers, hemolysis panel, and trace element analysis, with reference ranges.

Parameters	Results	Reference range
Vital signs		
Heart rate (beats per minute)	78	60-100
Blood pressure (mmHg)	118/72	90-120/60-80
Respiratory rate (breaths per minute)	16	12-20
Temperature (°C)	36.8	36.1-37.2
Oxygen saturation (%)	98	≥95
Complete blood count		
Hemoglobin (g/dL)	6.0	12.0-16.0
Hematocrit (%)	18.5	36-46
Mean corpuscular volume (fL)	90	80-100
White blood cell count (×10³/µL)	5.8	4.0-11.0
Platelet count (×10³/µL)	210	150-400
Iron studies		
Serum iron (µg/dL)	92	50-170
Ferritin (ng/mL)	680	15-150
Total iron-binding capacity (µg/dL)	260	250-450
Transferrin saturation (%)	35	20-50
Nutritional studies		
Vitamin B12 (pg/mL)	1713	200-900
Folate (pg/mL)	>2684	140-628
Hemolysis panel		
Lactate dehydrogenase (U/L)	180	135-225
Haptoglobin (mg/dL)	110	30-200
Total bilirubin (mg/dL)	0.7	0.2-1.2
Trace elements		
Serum copper (µg/dL)	38	70-140

Despite receiving erythropoietin 20,000 units three times weekly and IV iron therapy, her hemoglobin level did not improve. Hematology consultation considered nutritional deficiency due to prior bariatric surgery [2]. Serum copper was 38 µg/dL (reference range: 70-140 µg/dL), confirming copper deficiency [5]. The patient received intravenous copper replacement during hospitalization, followed by oral copper supplementation after discharge. Four weeks later, hemoglobin improved from 6.0 to 10.0 g/dL, and she regained full functional mobility.

## Discussion

Copper deficiency is an underrecognized cause of cytopenias, particularly after Roux-en-Y gastric bypass surgery [2]. Copper plays a central role in iron metabolism through its function as a cofactor for ceruloplasmin and hephaestin, which are essential for intestinal iron export and mobilization of iron from tissue stores [6-9]. Copper is also required for erythrocyte longevity via superoxide dismutase activity [10,11]. Deficiency may therefore result in ineffective erythropoiesis despite adequate iron stores, neutropenia due to arrested granulocytic maturation [12-16], and ringed sideroblast formation within the bone marrow [11].

In addition to its role in iron handling, copper modulates differentiation and self-renewal of hematopoietic progenitor cells, influencing multilineage hematopoiesis [17]. As a result, copper deficiency can produce bone marrow findings that closely resemble myelodysplastic syndrome (MDS), including cytoplasmic vacuolization, dysplastic changes, and sideroblast formation [18,19]. Several reports have demonstrated complete reversal of marrow dysplasia and cytopenias following copper repletion, highlighting the importance of early recognition of this reversible condition [18,19].

Our patient’s dramatic hematologic and functional recovery following intravenous copper replacement underscores the importance of including copper deficiency in the differential diagnosis of transfusion-refractory anemia, particularly in patients with a history of bariatric surgery or malabsorptive conditions. Intravenous supplementation is preferred in cases of severe deficiency, followed by oral copper for maintenance therapy [20]. Periodic monitoring of copper levels in high-risk patients may help prevent severe hematologic and neurologic complications [2].

## Conclusions

Copper deficiency is a reversible cause of anemia and cytopenias. It should be considered in patients with unexplained anemia, history of bariatric surgery, or malabsorptive conditions. Early recognition and treatment are critical, especially in patients unable to receive transfusions.
